# Raman Signal Enhancement Tunable by Gold-Covered Porous Silicon Films with Different Morphology

**DOI:** 10.3390/s20195634

**Published:** 2020-10-02

**Authors:** Svetlana N. Agafilushkina, Olga Žukovskaja, Sergey A. Dyakov, Karina Weber, Vladimir Sivakov, Jürgen Popp, Dana Cialla-May, Liubov A. Osminkina

**Affiliations:** 1 Physics Department, Lomonosov Moscow State University, 119991 Moscow, Russia; shevchenko.svetlana@physics.msu.ru; 2 Institute of Physical Chemistry and Abbe Center of Photonics, Friedrich Schiller University Jena, Helmholtzweg 4, 07745 Jena, Germany; Olga.Zukovskaja@leibniz-ipht.de (O.Ž.); karina.weber@leibniz-ipht.de (K.W.); juergen.popp@ipht-jena.de (J.P.); dana.cialla-may@leibniz-ipht.de (D.C.-M.); 3 Research Campus InfectoGnostics, Philosophenweg 7, 07743 Jena, Germany; 4 Leibniz Institute of Photonic Technology, Member of the Leibniz Research Allicance, Leibniz Health Technologies, Albert-Einstein-Straße 9, 07745 Jena, Germany; 5 Skolkovo Institute of Science and Technology, Nobel Street 3, 143025 Moscow, Russia; s.dyakov@skoltech.ru; 6 Institute for Biological Instrumentation of Russian Academy of Sciences, 142290 Pushchino, Russia

**Keywords:** porous silicon, gold, surface-enhanced Raman scattering, plasmonics, sensorics

## Abstract

The ease of fabrication, large surface area, tunable pore size and morphology as well surface modification capabilities of a porous silicon (PSi) layer make it widely used for sensoric applications. The pore size of a PSi layer can be an important parameter when used as a matrix for creating surface-enhanced Raman scattering (SERS) surfaces. Here, we evaluated the SERS activity of PSi with pores ranging in size from meso to macro, the surface of which was coated with gold nanoparticles (Au NPs). We found that different pore diameters in the PSi layers provide different morphology of the gold coating, from an almost monolayer to 50 nm distance between nanoparticles. Methylene blue (MB) and 4-mercaptopyridine (4-MPy) were used to describe the SERS activity of obtained Au/PSi surfaces. The best Raman signal enhancement was shown when the internal diameter of torus-shaped Au NPs is around 35 nm. To understand the role of plasmonic resonances in the observed SERS spectrum, we performed electromagnetic simulations of Raman scattering intensity as a function of the internal diameter. The results of these simulations are consistent with the obtained experimental data.

## 1. Introduction

Raman spectroscopy is a traditional sensing technique to detect the presence of chemical substances, pollutants, toxins etc. Surface-enhanced Raman scattering (SERS) offers an effective technique to improve the efficiency of Raman detection [1,2]. The signal enhancement is mainly attributed to the electromagnetic or/and chemical mechanism which provide a significant SERS enhancement up to 10^14^ [2,3,4]. The sensitivity of SERS analysis allows to “fingerprint” even single molecules [5]. This advantage makes SERS a unique tool for detection and diagnostic assays. As shown earlier, SERS-active surfaces were suitable to detect pesticides [6], nutrition-related molecules [7,8], bacteria [9,10], tumors [11,12] and viruses [13].

The quality of SERS-active surfaces is usually characterized by an enhancement factor (EF). Usually, the EF depends on various parameters [14], and is often associated with so-called ‘hot spot’ formation. The reproducibility of Raman signals is one more important index to evaluate the efficiency of SERS-active surfaces that means that the surface should be homogenous. With this in mind, the search for a universal and highly efficient substrate still continues, developing well-known techniques and offering new fabrication techniques. The most common way to increase the efficiency of SERS-active surfaces is to use a nanoscaled surface modified with nanoparticles of noble metals. The plasmonic surfaces, which include solid substrate with metallic nanostructures and chemically synthesized noble metal colloids, and their fabrication methods were reviewed in a number of papers [15,16,17]. From another side, a number of publications have shown that different porous materials can be used as a matrix for SERS-active metals, for example: alumina [18], porous Si/SiO_2_ [19], porous silicon [20], silicon nanowires [9,21,22], porous titanium oxide [23] and porous silver [13]. As is well-known, the bio-friendly properties [24] of porous silicon have been proven to act as a biocompatible and biodegradable material [25,26], as shown in a number of papers related to its biomedical applications [27,28]. Thereby, porous silicon is an appropriate surface for the adhesion of different types of microbiological material [29,30,31]. The PSi fabrication process is usually based on electrochemical etching of monocrystalline silicon (c-Si) [32]. The characteristics of the initial c-Si and the etching parameters (etching time, current intensity, active etchant concentration and temperature) influence such basic properties of the porous layer as porosity, thickness, size and structure. Based on the diameter of pores, PSi can be classified as microporous, mesoporous and macroporous [28].

Nanostructures based on PSi with deposited metal nanoparticles have been shown the capabilities to detect different dyes. For instance, a detectable concentration as low as 100 pM of crystal violet has been achieved by using PSi surface covered with silver nanoparticles (Ag NPs) [33]. Additionally, 1 pM of R6G was detected by Ag NPs/PSi grating structure system [34]. The gold covered PSi surfaces exhibited an ability to detect R6G [35,36] and cyanine (Cy) dyes molecules [36]. PSi with Ag nanostructures on a top were also checked for enhanced Raman activity on a Cy, evidencing detectable concentrations as low as 10^−7^ M [37].

Focusing on SERS-detection of more complex molecules and structures, a number of works have been reported that PSi to be a rather suitable substrate [38,39,40]. One of the issues of PSi implementation as surfaces specifying the geometry of plasmon metals is to understand the EF dependence on the porosity rate of silicon surface. Kosovic et al. studied microporous (pore sizes < 2 nm) and macro-mesoporous PSi (pore sizes > 10 nm) covered by silver nanostructures where R6G molecules immerged onto the surface could be detected down to concentration of 10^−9^ M and 10^−15^ M, respectively, at a 514.5 nm laser excitation wavelength. It was assumed that macro-mesoporous substrate had much more ‘hot spots’ with the optimized morphology (silver film morphology was heterogeneous with broad distribution of sizes and shapes) to produce large enhancements, and hence, the probability of detecting R6G molecules was much higher [41]. As for mesoporous silicon, Zeiri et al. detected lower as 10^−12^ M concentration of R6G molecules using meso-PSi (pore diameter 15 nm) with plasmonic AgNPs at the same laser excitation wavelength of 514.5 nm [20]. Artsemyeva et al. as shown that the detection limit for R6G absorbed on Ni-Ag PSi substrate reached 10^−11^ M [42]. Unfortunately, all the above listed measurements cannot provide us with the correct explanation of the pore sizes influence on the quality of the SERS surfaces due to different sizes of plasmonic nanostructures, diverse methods of SERS measurements, distinct laser excitations etc.

In the present paper we evaluated the Raman signal enhancement as a function of the distance between Au NPs tunable by porous silicon films with different morphology. 4-mercaptopyridine (4-MPy) and Methylene blue (MB) were used to describe SERS activity of obtained Au/PSi samples. The electromagnetic field distribution of incident plane wave was simulated and the appearance of hot spots amplifying the Raman signal was demonstrated.

## 2. Materials and Methods

### 2.1. Fabrication of Porous Silicon Layer and Au/PSi Surfaces

PSi layers were prepared by electrochemical etching of a single crystalline highly boron doped (10^20^ cm^−3^) silicon wafer (с-Si) in the presence of hydrofluoric acid (HF) 49% and ethanol (C_2_H_5_OH) for 30 s with current intensity 50 mA/cm^2^ [28]. Different pore sizes of the layers were achieved by changing the ratio of HF : C_2_H_5_OH. Finally, a short pulse of the etching current with density 500 mA/cm^2^ was applied to detach the porous film from the crystal silicon substrate. Thereby, three types of PSi layers were obtained for HF : C_2_H_5_OH ratios: 3 : 1 (PSi-1); 1 : 1 (PSi-2) and 1 : 3 (PSi-3).

Gold coating of PSi layers (Au/PSi) was performed at room temperature by electroless wet-chemical deposition of Au nanoparticles on silicon nanocrystals by reducing of 0.02 M gold (III) chloride (AuCl_3_) in 5 M HF, taken in the ratio 1 : 1, for 20 s as described in our previous work [19]. Finally, the samples were rinsed several times in Millipore water and dried at room temperature.

### 2.2. Characterization of Au/PSi Samples

Carl Zeiss ULTRA 55 (Oberkochen, Germany) scanning electron microscopy (SEM) was chosen to characterize the surface morphology of the obtained samples.

Hydrophilicity and hydrophobicity of Au/PSi-1,2,3 was studied by contact angle measurements using Contact Angle System OCA from DataPhysics Instruments GmbH (Filderstadt, Germany). For this, drop of water (1 µL) was placed onto the surface of the samples and angle between solid-liquid interface was measured once equilibrium was reached. The SEM images (size 700 × 600 nm) of bare and gold-coated PSi samples were analyzed by using software program ImageJ. The specific surface area of the samples was measured by the Brunauer, Emmett and Teller (BET) technique using Micromeritics TriStar 3000 BET system (Micromeritics, Aachen, Germany) with the five-points method using static-volumetric gas (nitrogen) adsorption. The porous films before the measurements were detached from c-Si substrate by applying shot of 500 mA/cm^2^ current pulse in HF : C_2_H_5_OH solution, and then degassed by heating in vacuum at 200 °C for 4 h.

### 2.3. SERS Measurements

4-Mercaptopyridine (4-MPy) and Methylene blue (MB) powders were purchased from Sigma Aldrich (Merck KGaA, Darmstadt, Germany). Firstly, stock solutions of 10^−3^ M for MB and 5 × 10^−4^ M for 4-MPy were prepared in high-purity water by adding the appropriate amount of powder. Next, stock solutions were diluted to have final concentrations of 10^−5^, 10^−6^ and 10^−7^ M for MB and 5 × 10^−5^, 5 × 10^−6^ and 5 × 10^−7^ M for 4-MPy.

For the SERS measurements, the samples were incubated for 30 min in the as-prepared solutions of the analytes and then air dried. The SERS measurements were performed using commercially available WITec confocal Raman system (WITec GmbH, Ulm, Germany) equipped with a 785 nm laser. During the measurements a 300 lines/mm grating was used with a spectral resolution of ~5 cm^−1^. The same objective (Nikon 50 × 0.95 N.A. (Nikon, Tokyo, Japan)) was employed for focusing the laser beam on the sample and for collecting the backscattered light. The laser power at the surface of the sample was 1 mW. For every substrate, three different areas in the size of 12 × 12 µm were measured using 0.5 µm step; this resulted in 576 spectra per scan. Scan areas were chosen randomly being in significant distance from each other. Integration time for every spectrum was 1 s.

Data processing was performed using an own-developed algorithm in the programming language R [43]. During the data processing, spectra were background corrected using the Statistics-sensitive Non-linear Iterative Peak-clipping (SNIP) algorithm [42]. For spectral comparison, mean SERS spectra for each concentration were calculated using preprocessed, vector-normalized spectra of all scans and cut to the region of interest.

### 2.4. Numerical Simulations

Calculations of electric field of incident plane electromagnetic wave were performed using boundary element method (BEM) approach developed in [44] for solving Maxwell equations and implemented in MNPBEM toolbox (developed by Ulrich Hohenester and Andreas Trügler from the University of Graz, Austria) [45,46,47] for the simulation of metallic nanoparticles. Dielectric permittivity of gold and silicon are taken from literature databases [48,49].

## 3. Results and Discussion

### 3.1. Samples Characterization

**Figure 1:**
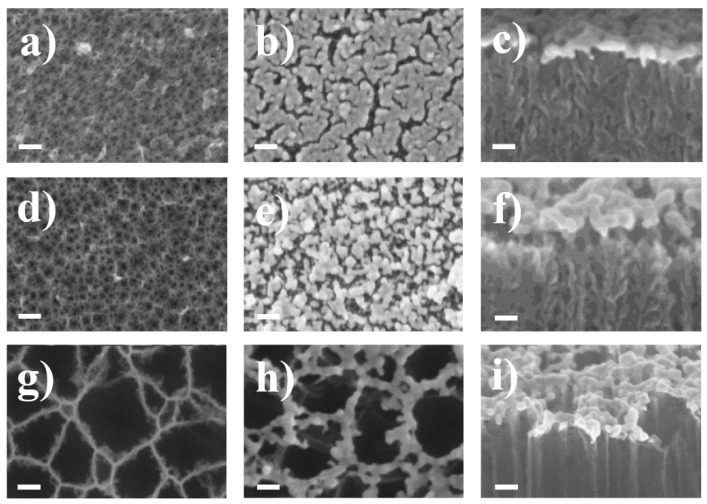
SEM planar and cross-section images of PSi films before (**a**,**d**,**g**) and after gold nanoparticle (Au NP) deposition (**b**,**c**,**e**,**f**,**h**,**i**): (**a**) PSi-1; (**b**) and (**c**) Au/PSi-1; (**d**) PSi-2; (**e**) and (**f**) Au/PSi-2; (**g**) PSi-3; (**h**,**i**) Au/PSi-3. Scale bar in all images is 50 nm.

Figure 1 shows planar images of the obtained PSi films with different pore sizes as well as planar and cross-sectional SEM images of the same samples after gold deposition (Au/PSi). The gold coating of PSi films was performed at room temperature by chemical deposition of Au nanoparticles (Au NPs) due to the reduction of gold (III) chloride (AuCl_3_) in HF [19].

The average porous layer thickness was 800 nm for PSi-1 and PSi-2 and 420 nm for PSi-3. No significant changes in porous layer thickness were observed after the Au NPs deposition. It can be seen from cross sectional SEM views, that Au NPs covered only the surface of the porous layer and do not penetrate into the pores, which can be explained by the charge states of the surface of the porous film.

Figure 1 clearly shows that the distance between the Au NPs is determined by the surface morphology of the PSi films. The smaller the pore diameter in the porous layer, the closer are the Au NPs. It is worth noticing that the distance between Au NPs for samples Au/PSi-1,2,3 is not equal to the PSi-1,2,3 pore diameters.

The pore diameter of PSi layers before gold coating as well as Au interparticle distance was estimated from SEM images using the ImageJ software (version 1.8.0; US National Institutes of Health, Bethesda, MD, USA). Pore diameter of PSi layers was also obtained by BET analysis. All values are summarized in Table 1. Note that PSi-1 and PSi-2 films with an average pore size of 15 and 20 nm, respectively, are considered mesoporous materials [28]. For PSi-3, the average pore size is 85 nm, and such a film is considered macroporous [28]. The diameter of Au NPs for all the samples was about 20 nm.

Unfortunately, it was impossible to study the PSi-3 layer by the BET method due to the impossibility of retaining the structure of this high porosity film after it was detached from the crystalline silicon substrate, as required in the experiment. However, as can be seen from Table 1, the average BET pore diameter for PSi-1 and PSi-2 correlates well with data obtained from SEM image analysis, and we can expect the same for PSi-3. The surface area of PSi films estimated to be 286 m^2^/g and 290 m^2^/g for PSi-1 and PSi-2, respectively.

**Figure 2:**
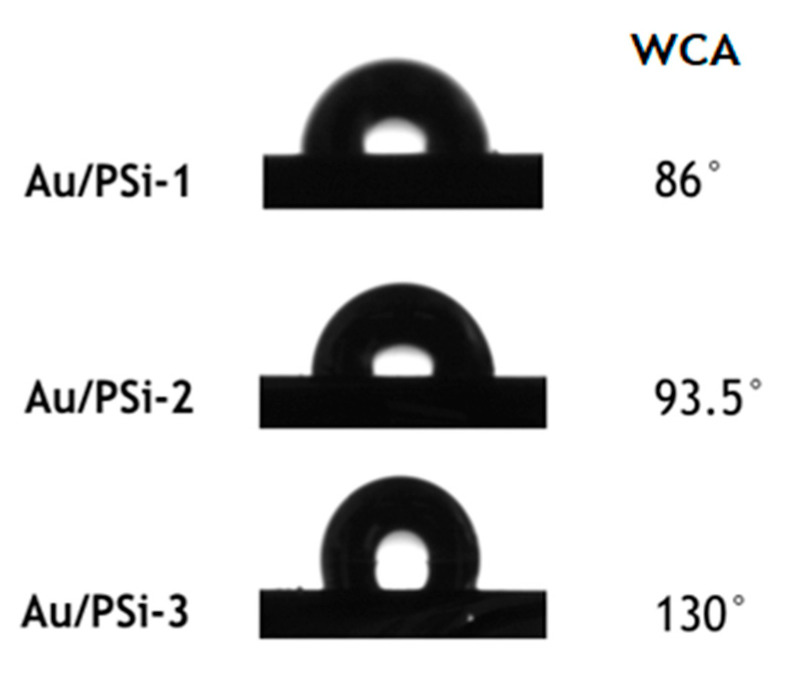
The water contact angle (WCA) of the drops placed onto the surface of Au/PSi-1,2,3.

In order to identify how pore diameter influences the hydrophilicity/hydrophobicity of the samples, and how the analyte molecules interact with Au NPs located on the PSi surface, contact angle measurements were performed. A drop of water (1 µL) was placed onto the surface of the obtained Au/PSi films, and the water contact angle (WCA) between solid-liquid interfaces was measured once equilibrium was reached (Figure 2).

From Figure 2 can be seen that sample with smaller pore size Au/PSi-1 was hydrophilic with the WCA 86°, with the increase of pore size samples became more hydrophobic with 93.5° for Au/PSi-2 and 130° for Au/PSi-3.

### 3.2. Results of Raman Spectroscopy Analysis

**Figure 3:**
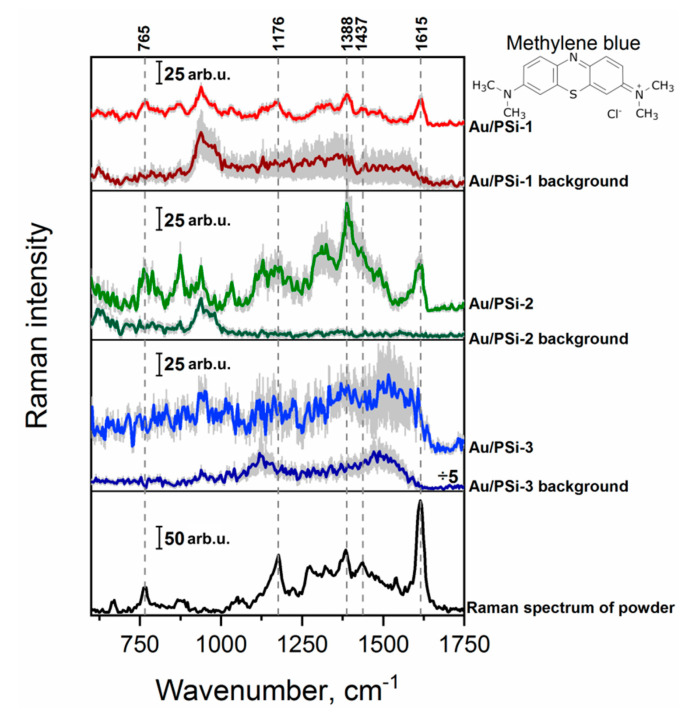
Raman spectrum of MB powder and mean SERS spectra of 10^−5^ M MB concentration on the surfaces with different porosity: Au/PSi-1, Au/PSi-2 and Au/PSi-3. Error scale bar (shown as grey shadow) for all presented spectra indicates the standard deviations from around 1500 measured spectra from three different areas of the substrate.

The SERS activity of the Au/PSi samples was investigated using two different target molecules: MB and 4-Mpy. MB is commonly in the industry used dye with a well-known SERS activity [42,50,51]. The Raman spectrum of the MB powder and SERS spectra of the lowest detectable MB concentration (10^−5^ M) on the films with different PSi porosity are presented in the Figure 3. The Raman mode at 765 cm^−1^ corresponds to the combined C–N–C and C–S–C skeletal deformation plus N–CH_3_ stretching, peak at 1176 cm^−1^ origins from the C–N stretching, the one at 1388 cm^−1^ is attributed to the in-plane ring deformation of C–N, while the signal at 1437 and 1615 cm^−1^ come from asymmetrical C–N stretching and from the C–C stretching of the ring, respectively [42,50,51]. These characteristic Raman bands were observed for the 10^−5^ M concentration of MB using surfaces Au/PSi-1 and Au/PSi-2. By comparison, no major bands of MB could be observed for macroporous sample Au/PSi-3. The highest signal intensity was registered for Au/PSi-2, while Au/PSi-1 provided more homogeneous signal. Keeping in mind some analytical applications, stability of the signal is often preferred over the intensity, thus, in this case preference would be given to Au/PSi-1 substrate. However, for the single molecule detection intensity is the most important factor, and here, Au/Psi-2 has higher potential.

**Figure 4:**
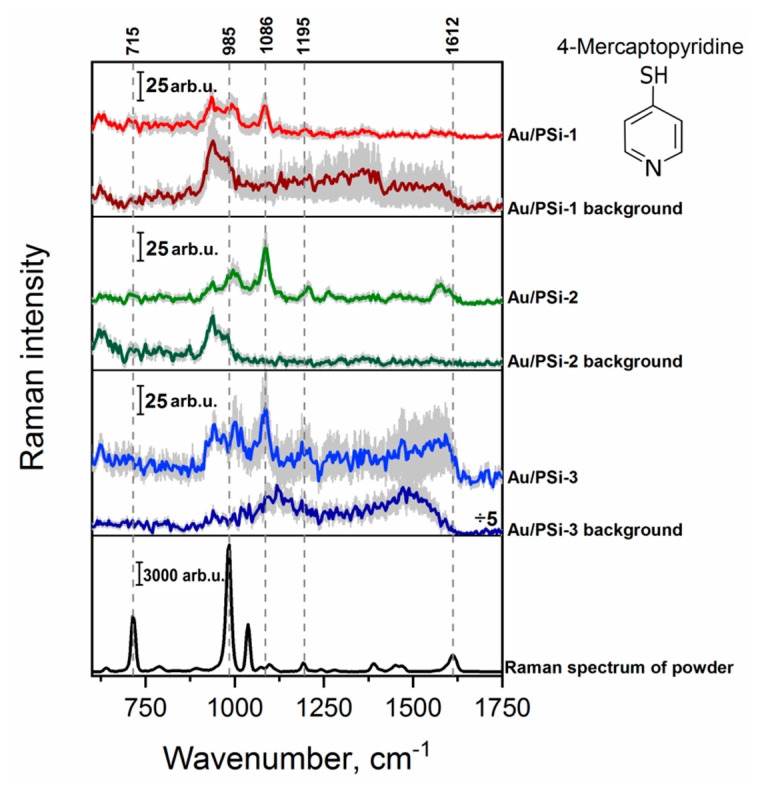
Raman spectrum of 4-Mpy powder and mean SERS spectra of 5 × 10^−6^ M concentration of 4-MPy on the surfaces with different morphology: Au/PSi-1, Au/PSi-2 and Au/PSi-3. Error scale bar (shown as grey shadow) for all presented spectra indicates the standard deviations from around 1500 measured spectra from three different areas of the substrate.

Next, 4-Mpy was investigated as a probe molecule. 4-Mpy is a typical aromatic thiol compound consisting of a thiol group in the position para to N atom in the pyridine ring. This special structure allows it to form well-ordered self-assembled monolayers on metal surfaces, which have great promising uses in many fields such as sensors, catalysts, and optics [51]. In Figure 4 the Raman spectrum of the powder and SERS spectra of the lowest detectable concentration of 4-MPy (5 × 10^−6^ M) on different surfaces are depicted. With all investigated films it was possible to record a SERS spectrum of 4-MPy. Here, the peak at 985 cm^−1^, attributed to the ring breathing mode, was enhanced and shifted to the higher wavenumbers. The most intensive peak in the SERS spectra at 1086 cm^−1^ corresponds to the C–H out-of-plane bend and the next one at 1195 cm^−1^ is assigned to the C–H with N–H bending [52,53]. The highest signal intensity here also was registered for Au/PSi-2. Moreover, smaller standard deviation indicates that this substrate is more homogeneous than two other ones.

**Figure 5:**
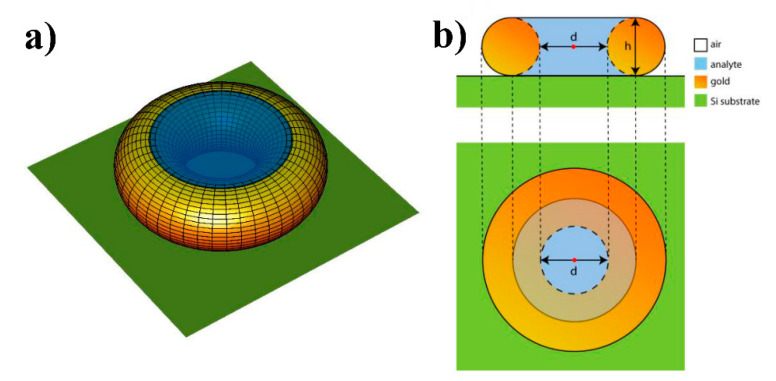
Schematic representation of the gold torus (**a**) showing the cross-sectional circles and planar section (**b**). The red dot in the middle of the torus in (**b**) denotes the coordinate where the electric field of incident plane wave is calculated.

Calculating the Raman signal intensity as a function of Au internal diameter we used the model of an Au torus placed at 3 nm above silicon substrate as schematically represented in Figure 5. In our calculations, we varied the internal diameter of the torus, d, and the diameter of a cross-sectional circle, h. The Au torus is illuminated by a normally incident plane wave with wavelength in vacuum λ = 785 nm. In our model, the Raman response is generated by the volume of analyte substance confined within the internal cavity of torus (Figure 5). We estimate the Raman signal intensity as:

(1) $$I_{R}\left(d,h\right)\sim \frac{V}{A}{\left|\vec{E}\left(d,h\right)\right|}^{4},$$

where $\vec{E}$ is the electric field of the incident plane electromagnetic wave taken at the center of the torus (red point in Figure 5b), A is the area occupied by the torus on the two-dimensional (2D) plane and V is the analyte volume. The Raman signal intensity calculated by Equation (1) is averaged over the orthogonal polarization states of incoming wave. From the electrodynamic point-of-view, this method of calculating the Raman signal intensity is analogous to the method of calculating the photoluminescence intensity where the excitation and out-coupling efficiencies are calculated separately at different photon energies and wavevectors [54]. In the case of Raman signal intensity, we neglect by difference between the energies of excitation and scattered light due to the small Stokes shift. Moreover, in our model we consider the excitation and collection angles to be zero that enables us to use Equation (1); however, inclined angle corrections can be done. We note that such a naive model does not account for many facts such as a discrete character of the scattering volume, real size of molecules, geometry imperfections etc. From the other side, the internal diameter of pores can be interpreted as the interparticle distance, and, as will be evident below, this model enables us to explain the experimental fact that there is an optimal Au internal diameter which provides the maximal Raman signal enhancement.

**Figure 6:**
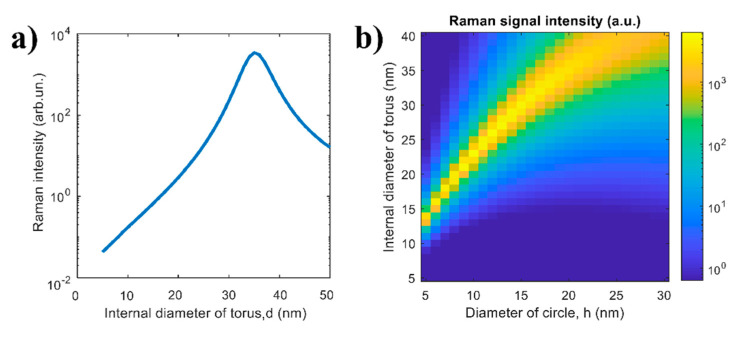
Theoretical dependence of the Raman signal intensity (**a**) on the internal diameter of torus *d* calculated as *h* = 20 nm and (**b**) on both parameters *d* and *h*.

The Raman intensity as a function of the internal diameter of torus d calculated by Equation (1) at *h* = 20 nm is shown in Figure 6a. One can see that there is an optimal value of the parameter *d* ≈ 35 nm, where the Raman signal is maximal. This is explained by the fact that with an increase of *d* the relative volume of analyte substance increases while the average electric field at the position of analyte molecules decreases. This situation is also observed for larger diameters of circle, *h* (Figure 6b); however, the optimal value of the parameter d increases. The considered example argues that the presence of the optimal internal diameter for the Raman signal intensity can be explained and understood from the optical viewpoint.

## 4. Conclusions

The presented results show that the Raman signal enhancement strongly depends on the internal diameter of torus-shaped plasmonic nanoparticles, which can be easily controlled by depositing them on porous silicon films with different morphologies. Methylen blue (MB) and 4-mercaptopyridine (4-MPy) were used to describe SERS properties of Au/PSi and to estimate the input of pore size and distance between Au NPs into the SERS enhancement factor. For the investigated molecules, the Au/PSi-2 surfaces with average Au torus internal diameter of 35 nm have shown the best SERS activity.

We have modeled the enhancement of Raman signal from analyte in the proximity of Au NPs. The theoretical internal diameter dependence of the Raman intensity has a single maximum, which results from the contribution of two factors. The first factor is the increase of analyte volume with the increase of the internal diameter; the second factor is the decrease of the average electric field of excitation wave at the position of analyte molecules with increase of interparticle distance. The simulation of the Raman intensity behavior correlates well with the experimental results.

The results have shown that the Raman signal enhancement and quenching can be tuned by morphology of the Au/PSi substrate, which is important for their use as a SERS-active substrate for molecular sensing.

## Figures and Tables

**Table 1:** The average pore diameter of PSi films obtained from SEM and BET analysis, values of the BET surface area of the samples and from SEM estimated Au NPs interparticle distance.

Measured Parameter	PSi-1	PSi-2	PSi-3
SEM average pore diameter	15 ± 5 nm	20 ± 5 nm	85 ± 15 nm
BET average pore diameter	16 ± 2 nm	21 ± 2 nm	–
BET surface area	286 m^2^/g	290 m^2^/g	*–*
SEM average Au NPs interparticle distance	21 ± 8 nm	30 ± 6 nm	65 ± 27 nm
